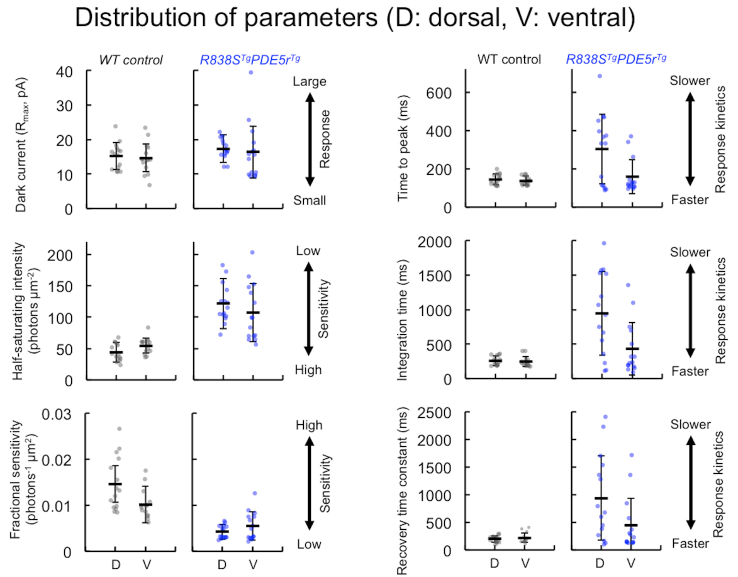# Phosphodiesterase 5 expression in photoreceptors rescues retinal degeneration induced by deregulation of membrane guanylyl cyclase

**DOI:** 10.1016/j.jbc.2025.108265

**Published:** 2025-02-03

**Authors:** Alexander M. Dizhoor, Shinya Sato, Zhuokai Luo, Lyuqi Tan, Fay E. Levin, Elena V. Olshevskaya, Igor V. Peshenko, Vladimir J. Kefalov

**Affiliations:** 1 Pennsylvania College of Optometry, Salus at Drexel University, Elkins Park, Pennsylvania, United States; 2 Graduate Program in Biomedicine, Salus at Drexel University, Elkins Park, Pennsylvania, United States; 3 Department of Neurobiology and Anatomy, Drexel University, Philadelphia, Pennsylvania, United States; 4 Gavin Herbert Eye Institute, Department of Ophthalmology and Center for Translational Vision Research, University of California, Irvine, California, United States; 5 Department of Physiology and Biophysics, University of California, Irvine, California, United States

**Keywords:** calcium binding proteins, cyclic GMP (cGMP), eye, GCAP, guanylate cyclase (guanylyl cyclase), *GUCA1A*, *GUCY2D*, photoreceptor, RetGC, retinal degeneration, PDE5, phosphodiesterase

## Abstract

Mutations in retinal membrane guanylyl cyclase 1 (RetGC1) and its calcium-sensor protein (guanylyl cyclase activating protein 1, GCAP1) cause congenital dominant retinopathies by elevation of cGMP synthesis in photoreceptors in the dark. We explored counteracting the elevated cGMP synthesis causing photoreceptor degeneration using ectopic expression of a nonphotoreceptor cGMP phosphodiesterase (PDE) isozyme PDE5. PDE5 primary structure was modified to direct the delivery of the recombinant PDE5 (PDE5r) to rod outer segments, by placing a C-terminal fragment derived from a cone-specific alpha-subunit of PDE6C at the C terminus of the PDE5, which allowed PDE5r expressed under control of mouse rod opsin promoter to accumulate in rod outer segments. Expression of PDE5r did not affect calcium-sensitivity of RetGC regulation in *PDE5r*^*Tg*^ transgenic retinas, but increased cGMP hydrolysis in the dark, which partially desensitized *PDR5r*^*Tg*^ rods in the dark *via* an “equivalent light” effect, analogous to exposure to a constant dim light of ∼20 to 40 photons μm^−2^ sec^−1^. The calcium-sensitivity of RetGC regulation remained drastically shifted outside the normal physiological range in hybrid *R838S*^*Tg*^*PDE5r*^*Tg*^ rods expressing both PDE5r and R838S RetGC1, the mutant causing *GUCY2D* dominant retinopathy, but the hybrid rods demonstrated a dramatic rescue from degeneration caused by the R838S RetGC1. In a similar fashion, PDE5r expression rescued degeneration of rods harboring Y99C GCAP1, one of the GCAP1 mutants most frequently causing *GUCA1A* dominant retinopathy. Our results open a possibility that ectopic expression of PDE5 can be used as an approach to rescue presently incurable dominant *GUCY2D* and *GUCA1A* retinopathies at the expense of a moderate reduction in rod light-sensitivity.

Cyclic GMP mediates rod and cone responses to light by regulating permeability of the cGMP-gated cyclic-nucleotide gated channels (CNG) in plasma membrane of rod and cone outer segments (reviewed in (1, 2, 3, 4)). Two processes regulate cGMP levels in photoreceptors: (i) activation of its hydrolysis by rhodopsin-transducin-phosphodiesterase-6 cascade in the light (reviewed in (4, 5)), and (ii) regulation of cGMP synthesis by negative calcium-feedback (1, 2, 5, 6). The negative calcium-feedback on retinal membrane guanylyl cyclase (RetGC) is mediated by guanylyl cyclase activating proteins (GCAPs). GCAPs decelerate RetGC activity in the dark, when Ca^2+^ entering *via* CNG channels converts GCAPs into a Ca^2+^-liganded state and, conversely, they accelerate it after illumination, when Ca^2+^ influx through the channels stops and GCAPs become converted into the Mg^2+^-liganded, “RetGC-activator” state (1, 5, 6). Whereas calcium-feedback suppresses fast cGMP production at the normal (high) Ca^2+^ concentrations in dark-adapted photoreceptors (7, 8), mutations in RetGC1 isozyme coded by *GUCY2D* gene (or in GCAP1 coded by *GUCA1A*) alter calcium-sensitivity of the RetGC1:GCAP1 complex (5, 9). This makes RetGC1 activity less sensitive to deceleration by calcium and elevates free cGMP levels in the dark (10, 11, 12). Replicating in mouse models mutations causing dominant human *GUCY2D* and *GUCA1A* retinopathies, such as dominant cone-rod dystrophy and cone- or cone-rod degenerations, directly demonstrate that elevation of the free cGMP and free Ca^2+^ concentrations by deregulated cGMP synthesis in the dark creates a “phototransduction disease” triggering photoreceptor death in these dominant retinopathies (10, 11, 12, 13, 14).

The dominant retinopathies caused by deregulation of cGMP synthesis are incurable forms of inherited blindness (9, 15, 16, 17, 18, 19, 20, 21, 22) that present a challenge for developing approaches based on gene therapy. One of the currently explored paradigms is gene editing designed to disable the disease-causing mutant alleles. The complexity of this approach is in difficulties to achieve the selectivity of disabling the mutant allele, which would require ablation of both the mutant and the WT allele simultaneously with transgenic delivery of a modified *GUCY2D* complementary DNA (cDNA) coding for a normal RetGC1 isozyme insensitive to gene ablation (23).

In the present study, we tested a different approach, based on the hypothesis that a stable acceleration of cGMP hydrolysis in the dark might counteract the accelerated production of cGMP by deregulated RetGC1:GCAP1 complex. This would reduce the free cGMP in the dark to levels tolerated by photoreceptors and thus rescue them from the degeneration. We further thought that it could be beneficial to use a nonphotoreceptor cGMP phosphodiesterase 5 (PDE5) to accelerate cGMP hydrolysis instead of trying to overexpress PDE6 for several reasons. First, the maximal activity of PDE5 is substantially lower than PDE6 (24, 25, 26, 27). Activation of PDE6, a very high-velocity enzyme (24, 25, 26, 27, 28), in the dark poses a risk that it can overly desensitize photoreceptors by closing all their CNG channels, thus making them drastically dysfunctional (11). Second, PDE6 is a multisubunit enzyme, and PDE6 maturation in photoreceptors requires a complex chaperoning process (29, 30, 31, 32), which has a propensity to destroy photoreceptors when adversely affected (30). Third, overexpression of PDE6 could alter phototransduction *per se*, which would add another layer of complexity to the expected outcome of the potential gene therapy.

We demonstrate here that the overexpression of PDE5 in mouse retinas harboring mutant RetGC1 or GCAP1 that case dominant retinopathies drastically reduces the extent of photoreceptor loss at the expense of a moderate decrease in light-sensitivity. We argue that this paradigm deserves further exploration for a potential use in gene therapy of *GUCY2D* and *GUCA1A* dominant retinopathies.

## Results

### Recombinant PDE5 accumulates in transgenic rod outer segments

**Figure 1:**
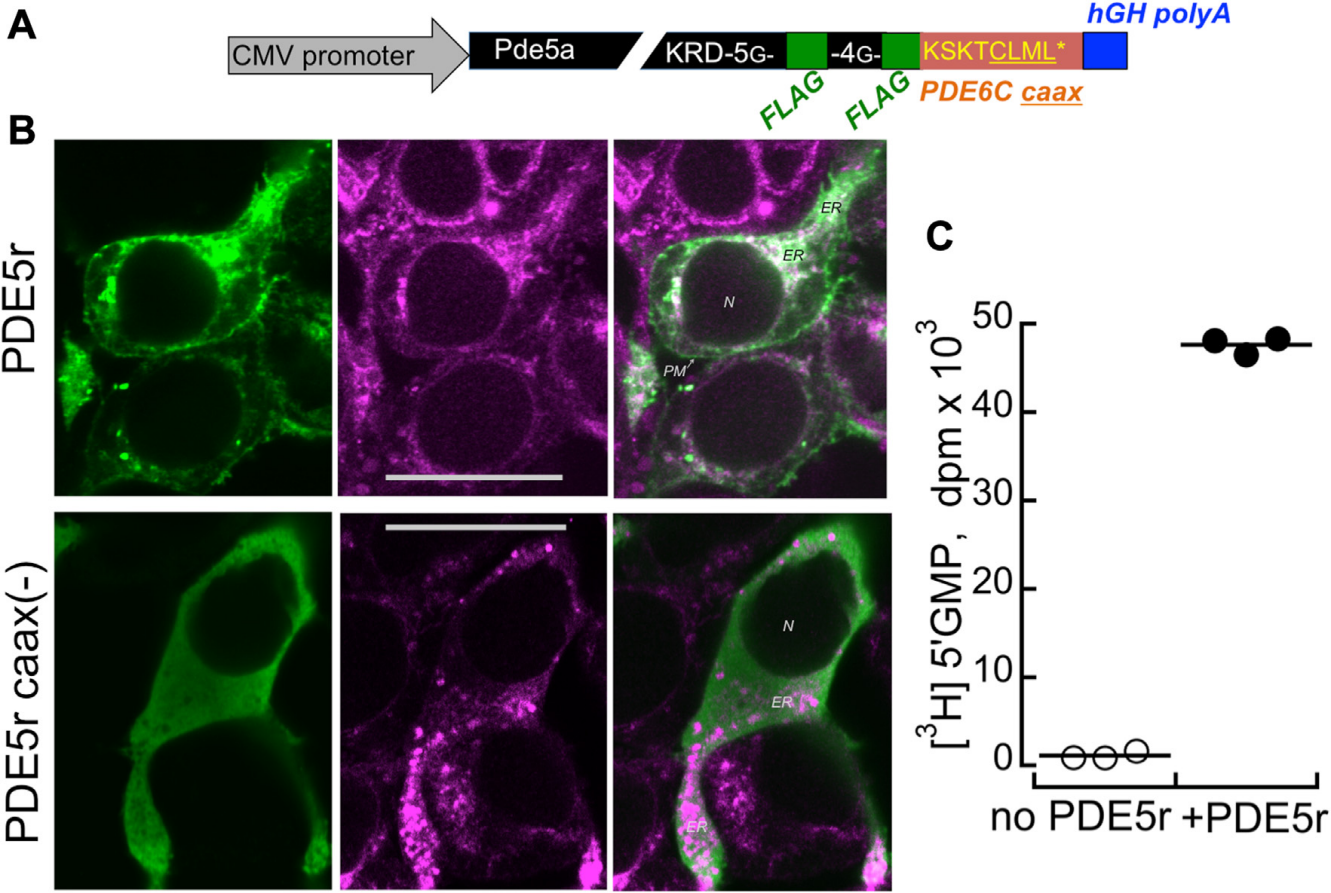
**Producing and testing PDE5r in HEK293 cells.***A*, the construct for PDE5r expression assembled in pCMV6 vector included CMV promoter, a cDNA for a mouse PDE5 (including its Kozak ribosome recognition motif) with the added coding sequences for duplicated FLAG epitopes connected *via* oligo-Gly linkers and eight C-terminal amino acid residues derived from a human PDE6C alpha subunit containing its isoprenylation signal, followed by human growth hormone gene fragment containing polyadenylation signal. *B*, comparison of the typical cellular distribution of PDE5r and PDE5r lacking the isoprenylation signal (“PDE5r caax-”) in HEK293 cells transfected with the respective construct; anti-FLAG fluorescence (*left panels*, *green*), ER-tracker fluorescence (*middle*, *pseudo-magenta*), merged images (*right*); the scale bar represents 20 μm; ER, endoplasmic reticulum, N, nucleus, PM, plasma membrane. *C*, hydrolysis of cGMP in homogenates of nontransfected (◯) and PDE5r-transfected (●) HEK293 cells; the activity was assayed in technical replicates as described under “Experimental procedures”. cDNA, complementary DNA; CMV, cytomegalovirus; HEK293, human embryonic kidney-293; PDE, phosphodiesterase; PDE5r, recombinant PDE5.

PDE6 accumulation in photoreceptor outer segments requires isoprenylation of its catalytic subunits (33), a modification that PDE5 is lacking (25). Therefore, a short C-terminal fragment derived from PDE6Cα was added to direct transgenic PDE5 produced by photoreceptors to the outer segment. PDE5 was modified by adding a dual FLAG epitope peptide followed by the eight-amino acid residue C-terminal peptide derived from human PDE6Cα subunit, KSKTCLML, containing CAAX isoprenylation signal (25, 33). The resultant recombinant PDE5 (PDE5r) (Fig. 1*A*) and a control “PDE5rCAAX(−)“ construct (also tagged by FLAG but lacking the PDE6Cα CAAX box) was expressed in transfected human embryonic kidney-293 (HEK293) cells using a cytomegalovirus (CMV) promoter and visualized by immunofluorescence using anti-FLAG antibody (Fig. 1*B*). Without the isoprenylation signal, PDE5 was primarily localized in cytosol. In contrast, the complete PDE5r construct harboring the PDE6Cα CAAX signal primarily associated with membrane structures, such as the plasma membrane and the endoplasmic reticulum. The presence of catalytic activity in recombinant PDE5r expressed in transfected cells was verified using *in vitro* assay (Fig. 1*C*).

**Figure 2:**
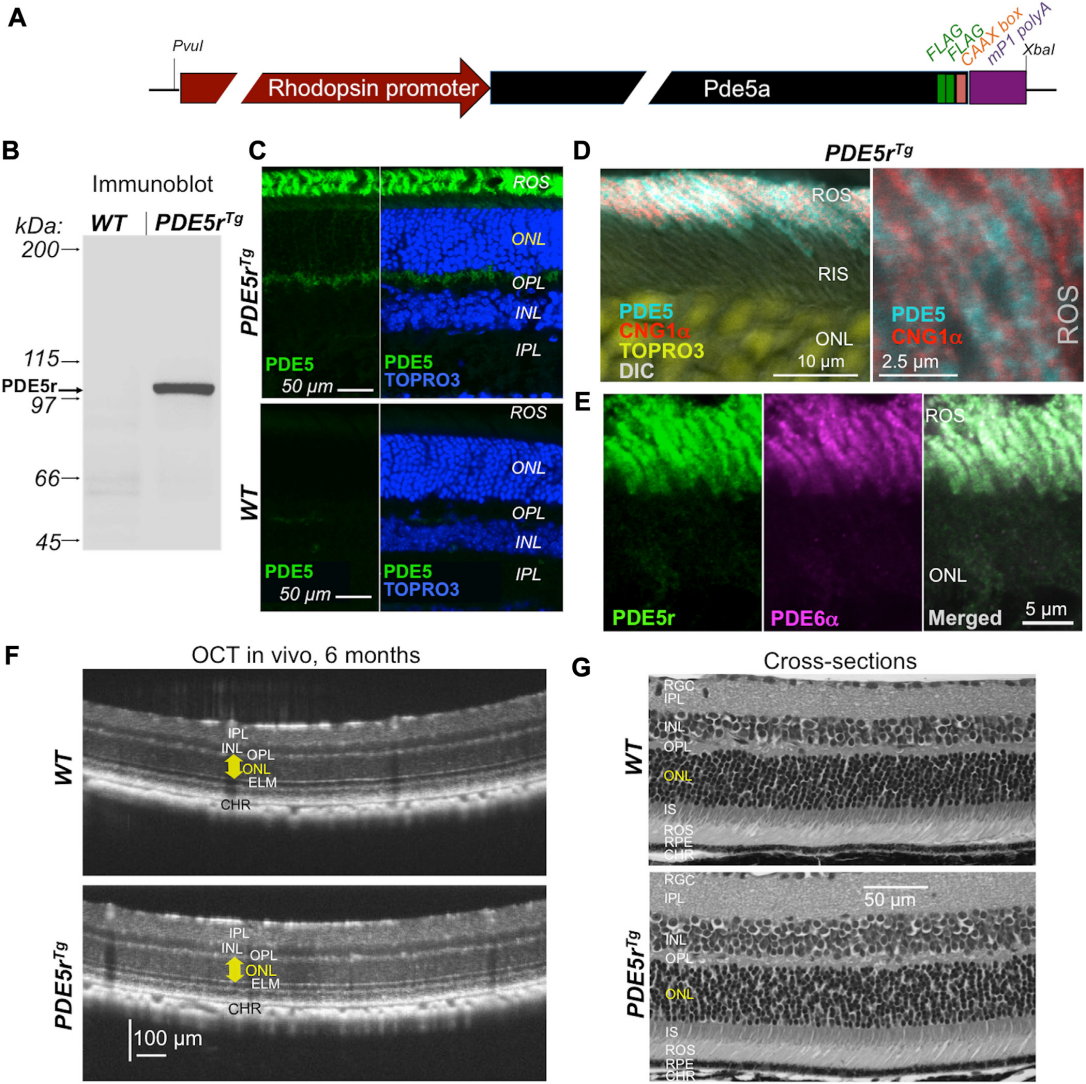
**Ectopic expression of PDE5r in a mouse retina.***A*, the PDE5r cDNA from pCM6 vector was transferred to a plasmid for the transgenic expression in mouse rods. The resultant plasmid harbored 4.2 kbp mouse rhodopsin promoter, PDE5r construct containing PDE5α cDNA, the two FLAG epitopes and the CAAX box as shown in Figure 1*A*, and mouse mP1 protamine gene fragment containing polyadenylation signal. The *PvuI/XbaI* fragment excised from the vector was used for random DNA insertion in C57BL/6J mouse pronuclei to produce F_0_ founders. *B*, immunoblotting of PDE5r in mouse retinas, line #22. Retinal extracts from *PDE5r*^*Tg*^ and WT sibling were analyzed by Western immunoblotting probed by anti-PDE5A antibody as described under “Experimental procedures”. *C*, immunofluorescence of PDE5r probed using anti-FLAG antibody (*green*) in cryosections of *PDE5r*^*Tg*^ (*upper panel*) and a WT sibling (*bottom* panel); in the *right* part of each panel, nuclei in the same image frame are counterstained with TO-PRO-3 (*pseudo-blue*). *D*, *Left* panel. PDE5r immunofluorescence (*pseudo-cyan*) in rod outer segments; counterstained by anti-CNG1α antibody (*red*) to mark ROS plasma membrane and TOPRO3 (*pseudo-yellow*) to mark nuclei. *Right* panel. *PDE5r*^*Tg*^ ROS are shown under higher magnification; PDE5r (*pseudo-cyan*) counterstained by anti-CNG1α antibody (*red*). *E*, PDE5r localization in ROS in comparison with PDE6. *PDE5r*^*Tg*^ retina section was probed with anti-FLAG mouse antibody to mark PDE5r localization (*left* panel, *green fluorescence*) and anti-PDE6α rabbit antibody (*middle* panel, *pseudo*-*magenta*); the merged images are shown in the rightmost panel. *F* and *G*, retinal morphology in WT and *PDE5r*^*Tg*^ mouse retinas at 6 months of age. *F*, representative optical coherence tomography (OCT) images of WT and *PDE5r*^*Tg*^ mouse retinas. *G*, representative hematoxylin/eosin-stained aldehyde-fixed retinal cross-sections of WT and *PDE5r*^*Tg*^ mouse retinas. Here and further the retinal histological layers are marked as follows: cDNA, complementary DNA; CHR, choroid; CNG, cyclic-nucleotide gated channel; ELM, external limiting membrane; INL, inner nuclear layer; IPL, inner plexiform layer; IS, rod inner segment layer; ONL, outer nuclear layer; OPL, outer plexiform layer; PDE, phosphodiesterase; PDE5r, recombinant PDE5; RGC, retinal ganglion cell layer; ROS, rod outer segment layer, RPE, retinal pigment epithelium.

Once the catalytic activity in PDE5r was confirmed, the same PDE5r DNA construct was expressed in transgenic mice under control of rod opsin promoter instead of CMV promoter (Fig. 2*A*). Immunoblotting using anti-PDE5 antibody showed PDE5r transgene expression in the retina (Fig. 2*B*), where it was located predominantly in rod outer segments (ROS) (Fig. 2, *C*–*E*), and more specifically—in the area containing the stack of photoreceptor disks of ROS counterstained with anti-CNG1α antibody (14) marking the plasma membrane of the outer segments (Fig. 2*D*). Localization of PDE5r in ROS appeared indistinguishable from that of PDE6, the endogenous soluble ROS enzyme associated with the photoreceptor disks as a peripheral membrane protein (Fig. 2*E*). Accumulation of PDE5r in the outer segments did not cause evident abnormalities in retinal morphology in living *PDE5r*^*Tg*^ mice aged 6 months when assessed by optical coherence tomography (OCT) or in postmortem retinal histological cross sections (Fig. 2, *F* and *G*).

### Change in light-sensitivity of PDE5^Tg^ transgenic mice

**Figure 3:**
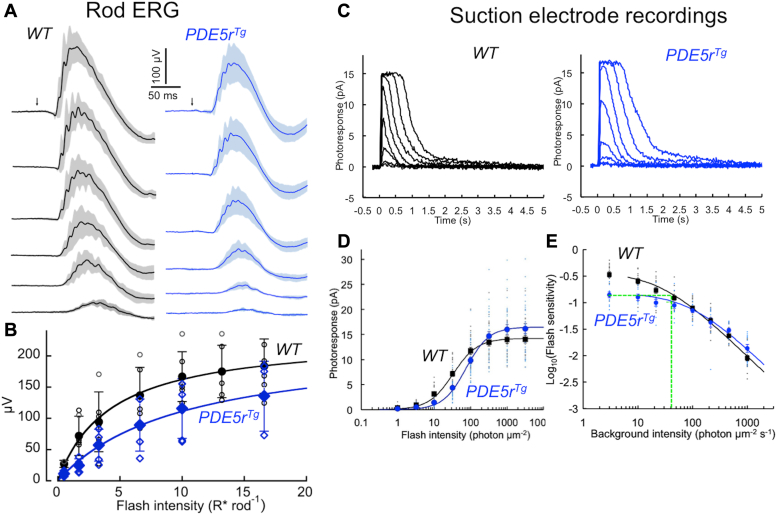
**Decreased light-sensitivity of *PDE5r***^***Tg***^**rods.***A*, rod scotopic ERG b-wave evoked by series of 505 nm flashes (*arrow*) delivering ∼0.5 to 17 photoisomerizations (R∗) per rod, averaged from five WT (●) and five *PDE5r*^*Tg*^ () mice (*solid line*: mean average; shaded area: ± SD). *B*, rod scotopic b-wave amplitudes averaged from independent recordings from five WT (●) and five *PDE5r*^*Tg*^ () mice, mean ± SD error bars; superimposed on small open symbols showing individual data points for each mouse. *C*, examples of flash response families in WT and *PDE5r*^*Tg*^ single-rod suction electrode recordings. Flash stimuli: 500 nm green, 1 to 3200 photons μm^−2^. *D*, intensity-response curves from rods in WT (◼) and *PDE5r*^*Tg*^ () mice; mean ± SE error bars, independent measurements; superimposed on small symbols showing individual data points for each recording for 25 WT rods and 30 *PDE5r*^*Tg*^ rods. Data were fitted with *R*_*max*_*∗I*^*n*^*/(I*_*1/2*_^*n*^ *+ I*^*n*^*)* where *R*_*max*_ is maximum response amplitude (pA), *I* is flash intensity (photons μm^−2^), *I*_*1/2*_ is half-saturating flash intensity (photons μm^−2^), and *n* is Hill coefficient. Fitting parameters: R_max_ = 14.2, I_1/2_ = 32.9, and n = 1.30 for WT, R_max_ = 16.5, I_1/2_ = 75.0, and n = 1.38 for *PDE5r*^*Tg*^; parameters values are summarized in Table S1. *E*, background adaptation. Flash sensitivity of WT (◼) and *PDE5r*^*Tg*^ () rods exposed to background light. Plots, mean ± SEM, for 17 WT rods and 12 *PDE5r*^*Tg*^ rods were fitted with log10 (*S*_*fD*_.*I*_*o*_/(*I*_*o*_ + *I*)), where *I* is background intensity (photons μm^−2^ sec^−1^), *S*_*fD*_ is flash sensitivity in darkness (plots at 0), and *I*_*o*_ is background intensity required to decrease the sensitivity by a factor of 2; fitted using *I*_*o*_ = 26.7 for WT and *I*_*o*_ = 92.7 for *PDE5r*^*Tg*^; mean ± SE error bars, independent measurements; superimposed on small symbols showing individual data points. The flash sensitivity in dark-adapted *PDE5r*^*Tg*^ rods mimics adaptation to a constant 20 to 40 photons μm^−2^ sec^−1^ light (*green line*). Other conditions are described under “Experimental procedures”. ERG, electroretinography; PDE, phosphodiesterase; PDE5r, recombinant PDE5.

Dark-adapted electroretinography (ERG) responses to flash strengths ranging from 0.5 to 17 photoisomerizations (R∗) rod^−1^ indicated partial reduction of light-sensitivity of *PDE5r*^*Tg*^ rods (Fig. 3, *A* and *B*). A comparison of individual rod sensitivity in WT and *PDE5r*^*Tg*^ mice using suction electrode recordings (Fig. 3*C*) also showed that the transgenic rods became nearly 2.5-fold less sensitive to light (Figs. 3*D* and Table S1): the light intensity required for the half-maximal response (I_1/2_) increased from 33 to 75 photons μm^−2^ (*t* test *p* < 0.0001). The reduction in sensitivity of dark-adapted *PDE5r*^*Tg*^ rods was consistent with the possibility that PDE5r produced an “equivalent light” effect in the dark. Indeed, transgenic rods behaved as WT rods exposed to a mild constant background light of 20 to 40 photons μm^−2^ sec^−1^ (Fig. 3*E*).

### PDE5r rescues degeneration of rods expressing R838S RetGC1

**Figure 4:**
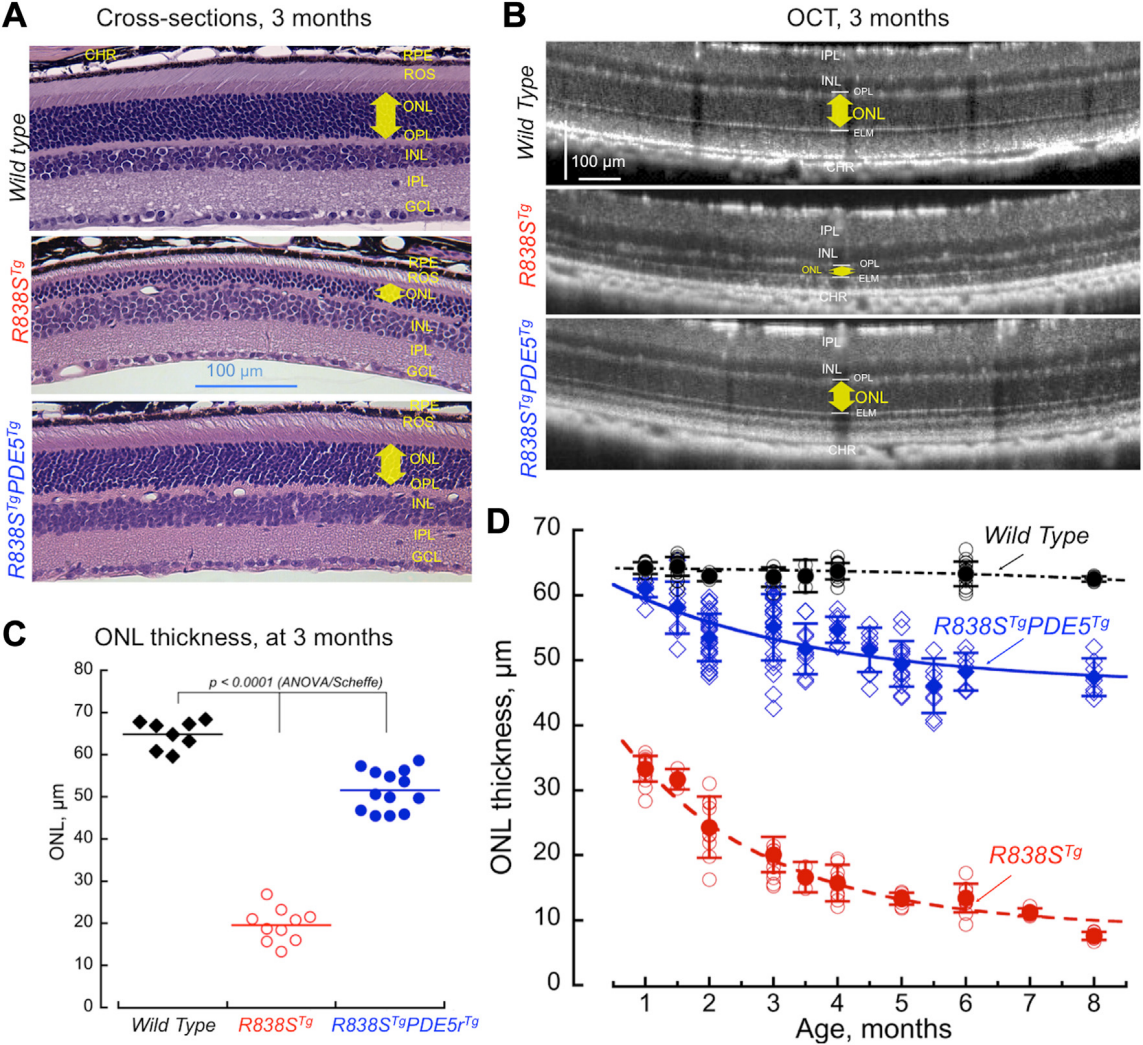
**PDE5r rescues degeneration of rods caused by R838S RetGC1.***A*, representative hematoxylin/eosin-stained aldehyde-fixed retinal sections from WT (*top*), *R838S*^*Tg*^ (*middle*), and *R838S*^*Tg*^*PDE5r*^*Tg*^ (*bottom*) mice aged 3 months. *B*, representative *in vivo* OCT imaging from WT (*top*), *R838S*^*Tg*^ (*middle*), and *R838S*^*Tg*^*PDE5r*^*Tg*^ (*bottom*) mice aged 3 months. The *yellow arrow marks* the thickness of ONL measured between OPL and ELM reflective layers; the OCT images are oriented with the choroid facing the *bottom*. *C*, outer nuclear layer thickness averaged by using independent *in vivo* OCT imaging from eight WT (◆), 10 *R838S*^*Tg*^ (), and 14 *R838S*^*Tg*^*PDE5r*^*Tg*^ () mice at 3 months of age; tested by ANOVA/Sheffe *post hoc*. *D*, reduction of ONL with age in WT (●◯), *R838S*^*Tg*^ ( ), and *R838S*^*Tg*^*PDE5r*^*Tg*^ ( ) mice; *open symbols*, OCT measurements in individual animals at the indicated ages; *filled symbols with error bars* (mean ± SD)–averaged ONL thickness at the indicated ages. ELM, external limiting membrane; OCT, optical coherence tomography; ONL, outer nuclear layer; OPL, outer plexiform layer; PDE, phosphodiesterase; PDE5r, recombinant PDE5; RetGC1, retinal membrane guanylyl cyclase 1.

*PDE5r*^*Tg*^ mice were crossed with transgenic mouse line (*R838S*^*Tg*^ line 379) expressing human R838S RetGC1, one of the previously characterized models harboring RetGC mutant that cause dominant cone-rod dystrophy in humans (34). In that mouse model, degeneration of the *R838S*^*Tg*^ transgenic rods is triggered by deregulation of Ca^2+^-sensitivity of RetGC1 leading to the elevation of both free cGMP and free Ca^2+^ in the outer segment (12, 34). By 3 months of age, *R838S*^*Tg*^ rods presented evidence of undergoing severe degeneration, whereas the retinal morphology in their littermates additionally harboring PDE5r looked near normal (Fig. 4, *A* and *B*). The progression of photoreceptor degeneration was drastically different between *R838S*^*Tg*^ and *R838S*^*Tg*^*PDE5r*^*Tg*^ mice, when compared in the same parts of the retinas. Rods expressing R838S RetGC1 degenerate fast (Fig. 4, *C* and *D*), such that only half of the rod nuclei remained after 1 month, and by 6 months of age the outer nuclear layer comprised by photoreceptor nuclei, in mice primarily rod nuclei (35), was nearly undetectable. In a striking contrast to that, the hybrid *R838S*^*Tg*^*PDE5r*^*Tg*^ mice retained complete complement of the photoreceptor nuclei at young age and nearly 70% of the photoreceptor nuclei at 6 months.

### cGMP synthesis *versus* hydrolysis in R838S RetGC1^Tg^PDE5r^Tg^ transgenic mice in the dark

**Figure 5:**
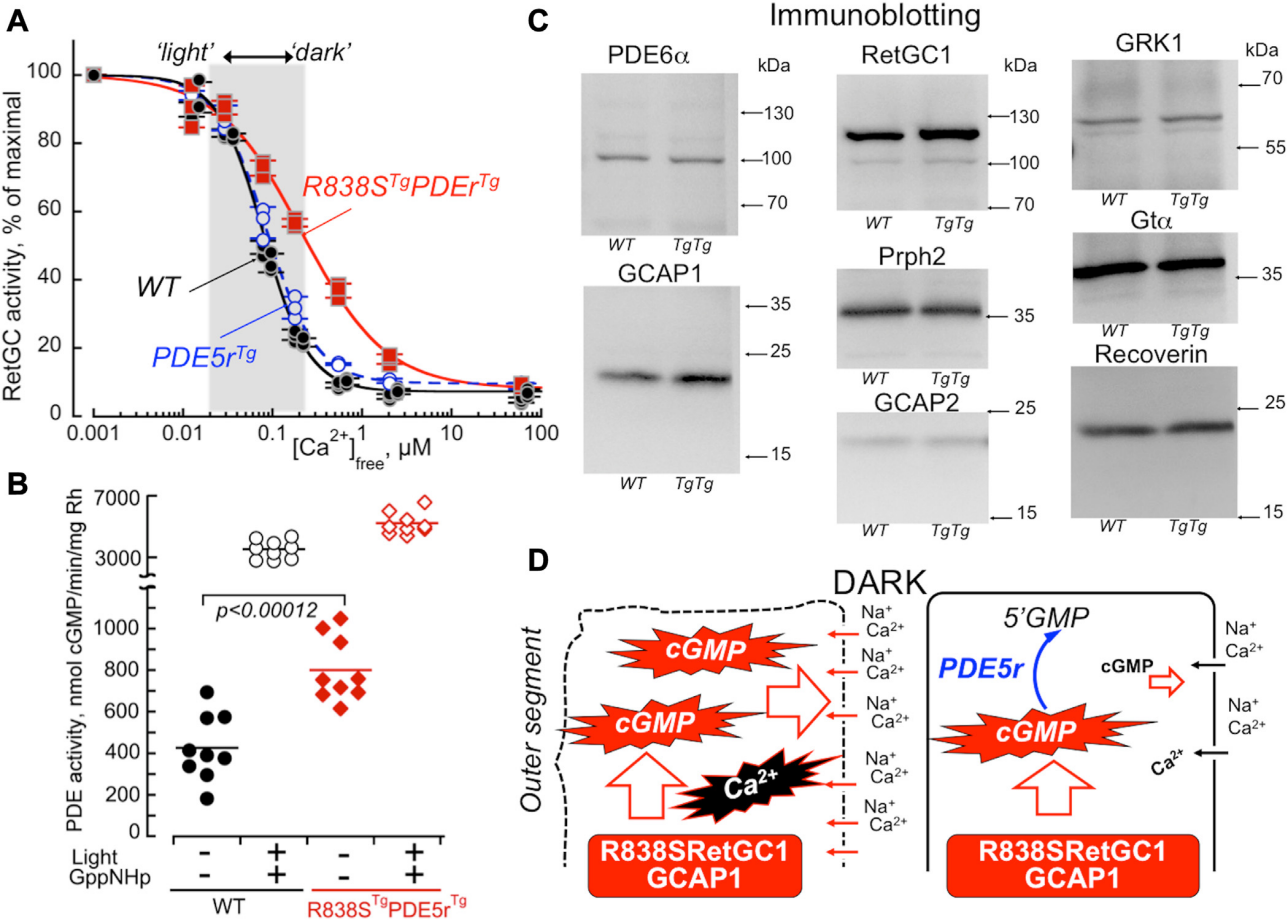
**Regulation of cGMP in transgenic retinas.***A*, Ca^2+^-sensitivity of cGMP synthesis (mean ± SD *error bars*) in WT (●) (n = 4), *PDE5r*^*Tg*^ () (n = 3), and *R838S*^*Tg*^*PDE5r*^*Tg*^ () (n = 3) different mouse retinas assayed as described under “Experimental procedures”. The *shaded area* approximates the change of free Ca^2+^ concentrations between light-adapted and dark-adapted mouse rod outer segments (7, 10). *B*, PDE activity in homogenates obtained from different dark-adapted WT (●◯) and *R838S*^*Tg*^*PDE5r*^*Tg*^ ( ) retinas in the dark in the absence of GppNHp (● ) or in the light in the presence of 0.2 mM GppNHp (◯ ); p–Student *t* test. *C*, Western immunoblotting of retinal samples from WT and *R838S*^*Tg*^*PDE5r*^*Tg*^ (TgTg) mice aged 3 months; the samples were equalized by rhodopsin content as described under “Experimental procedures”. *D*, the diagram of the expected change in cGMP flux in mouse rods harboring R838S RetGC1 *versus* hybrid rods harboring both R838S RetGC and PDE5r. Deregulated synthesis of cGMP by the mutant RetGC1 at normal concentrations of Ca^2+^ in dark-adapted photoreceptors elevates cGMP levels and increases influx of Ca^2+^ through the cGMP gated channels (12), thus creating the conditions provoking the onset of degeneration in *R838S*^*Tg*^ rods. Whereas the Ca^2+^ sensitivity of cGMP synthesis in *R838S*^*Tg*^*PDE5r*^*Tg*^ rods remains deregulated, elevation of cGMP hydrolysis in the dark by PDE5r brings the free cGMP levels closer to normal and reduces the abnormal influx of Ca^2+^, which helps offset the degeneration caused by the abnormal activity of R838S RetGC1 in the dark. GppNHp, guanylyl imidodiphosphate; PDE, phosphodiesterase; PDE5r, recombinant PDE5; RetGC1, retinal membrane guanylyl cyclase 1.

The dramatic rescue of *R838S*^*Tg*^*PDE5r*^*Tg*^ rods suggested that the abnormal cGMP production of *R838S*^*Tg*^ could be counteracted by the presence of PDE5r. We tested the regulation of cGMP in the rescued *R838S*^*Tg*^*PDE5r*^*Tg*^ retinas aged 3 months, the time when in *R838S*^*Tg*^ outer segments are mostly degenerated and therefore any biochemical heterogeneity in *R838S*^*Tg*^*PDE5r*^*Tg*^ rods due to possible cell-to-cell variability in PDE5r expression and the presence of rescued *versus* degenerating *R838S*^*Tg*^ rods would be minimized. Expression of PDE5r did not shift Ca^2+^-sensitivity of RetGC in WT strain background (Fig. 5*A*), and in the hybrid *R838S*^*Tg*^*PDE5r*^*Tg*^ mice, Ca^2+^-sensitivity of RetGC1 remained shifted outside the normal physiological range of Ca^2+^ in mouse rods in the dark (Fig. 5*A*), the abnormality causing *R838S*^*Tg*^ photoreceptor degeneration (12, 14). At the same time, the activity of cGMP hydrolysis in the dark in *R838S*^*Tg*^*PDE5r*^*Tg*^ retinas normalized per rhodopsin content was elevated approximately two-fold compared to the WT rods (Fig. 5*B*). Except for expressing R838S RetGC1 and PDE5r, the hybrid *R838S*^*Tg*^*PDE5r*^*Tg*^ showed a pattern of various phototransduction proteins expression that was similar to WT rods when standardized by rhodopsin content (Fig. 5*C*). Hence, in the *R838S*^*Tg*^*PDE5r*^*Tg*^ rods, the abnormally elevated cGMP production in the dark was counterbalanced by the elevated cGMP decay *via* PDE5r (Fig. 5*D*). This allowed most of the hybrid rods to survive over a long period of time, even at the ages when their littermates expressing R838S RetGC1 alone nearly completely lost their rods (Fig. 4).

### Light-sensitivity in R838S^Tg^PDE5r^Tg^ transgenic rods

**Figure 6:**
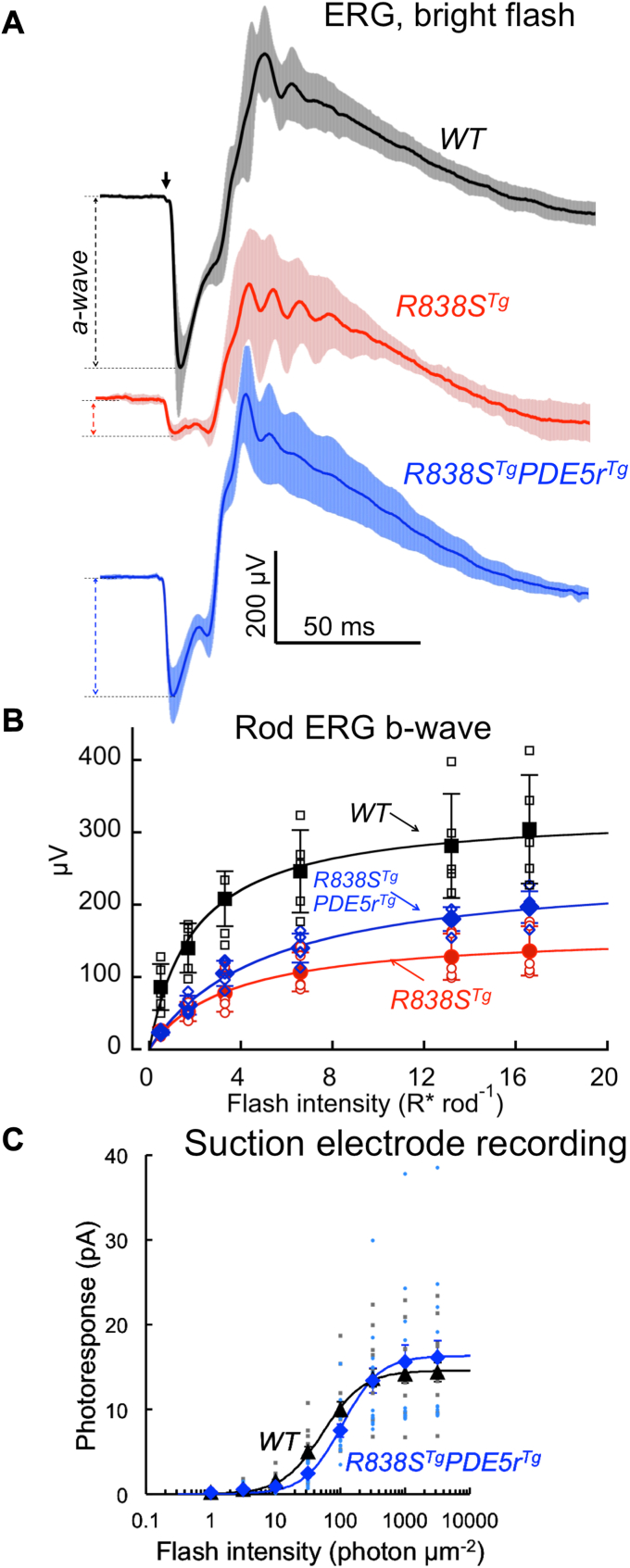
**Light sensitivity of the rescued *R838S***^***Tg***^***PDE5r***^***Tg***^**rods.***A*, ERG bright-flash (1 ms, 505 nm, ∼0.5 × 10^6^ R∗ rod^−1^) responses, mean (*solid black trace*) ± SD (*light shade*), in different dark-adapted WT (n = 7, *black*), *R838S*^*Tg*^ (n = 5, *red*), and *R838S*^*Tg*^*PDE5r*^*Tg*^ (n = 10, *blue*) mice aged 3 months. *B*, scotopic rod ERG b-wave amplitudes (mean ± SD) in dark-adapted WT (◼, n = 7), *R838S*^*Tg*^ (, n = 5), and *R838S*^*Tg*^*PDE5r*^*Tg*^ (, n = 10) mice to a flash strengths, ∼0.5 to ∼17 R∗ rod^−1^; superimposed on open symbols of the corresponding colors showing responses from different mice; the statistical significance of the differences from *R838S*^*Tg*^ mice (unpaired *t* test) were *p* < 0.01 in WT for all flash strengths and *p* < 0.05 in *R838S*^*Tg*^*PDE5r*^*Tg*^ for 13 and 17 R∗ rod^−1^. *C*, intensity-response curves in suction electrode recordings from ventral retina rods in WT (▲) and *R838S*^*Tg*^*PDE5r*^*Tg*^ () mice aged 3 months. Plots, mean ± SE error bars, superimposed on small symbols showing individual data points for each independent recording for 15 WT rods and 15 *PDE5r*^*Tg*^ rods, were fitted with *R*_*max*_*∗I*^*n*^*/(I*_*1/2*_^*n*^ *+ I*^*n*^*)* where *R*_*max*_ is maximum response amplitude (pA), *I* is flash intensity (photons μm^−2^), *I*_*1/2*_ is half-saturating flash intensity (photons μm^−2^), and *n* is Hill coefficient. Fitting parameters: R_max_ = 14.6, I_1/2_ = 54.5, and n = 1.38 for WT, R_max_ = 16.3, I_1/2_ = 107, and n = 1.49 for *R838S*^*Tg*^*PDE5r*^*Tg*^. ERG, electroretinography; PDE, phosphodiesterase; PDE5r, recombinant PDE5.

The *R838S*^*Tg*^*PDE5r*^*Tg*^ rods remained functional. In *R838S*^*Tg*^*PDE5r*^*Tg*^ mice, ERG a-wave amplitude to a bright flash (∼0.5 × 10^6^ photons/rod) at 3 months of age was markedly improved when compared to *R838S*^*Tg*^: 254 ± 60 μV (mean ± SD) (n = 10); *versus* 80 ± 19 μV (n = 5), respectively (Student *t* test *p* = 0.0004) (Fig. 6*A*). Evidently, the primary reason for this much better ERG preservation was a dramatic preservation of *R838S*^*Tg*^ rods by the ectopic expression of PDE5r. However, the ERG a-wave in *R838S*^*Tg*^*PDE5r*^*Tg*^ was still substantially reduced when compared to the WT 387 ± 90 μV (n = 7). Rod-specific scotopic ERG b-wave evoked by low-strength flashes, although improved in comparison with the *R838S*^*Tg*^, was substantially lower than in WT (Fig. 6*B*). Incomplete rescue of rods harboring R838S RetGC1 (Fig. 4*D*) due to possible cell-to-cell variations in PDE5r *versus* R838S RetGC1 expression could be responsible for the partial reduction of ERG. However, single-rod responses measured in *R838S*^*Tg*^*PDE5*^*Tg*^ using suction electrode recordings (Fig. 6*C*; Table 1) showed that their rod sensitivity itself was also altered, resulting in a moderate but well-detectable, two-to three-fold, reduction.

**Table 1:** Rod photoresponse parameters in suction electrode recordings^a^

Parameters	WT control					*R838S*^*Tg*^*PDE5r*^*Tg*^				
	Mean	SD	SE	n	P	Mean	SD	SE	n	P
Dark current, R_max_ (pA)										
Dorsal	15.2	3.88	1.00	15	0.697	17.2	2.96	0.77	15	0.680
Ventral	14.6	4.34	1.12	15		16.3	8.10	2.09	15	
Half-saturating Intensity, I_1/2_ (photons μm^−2^)										
Dorsal	43.9	13.7	3.54	15	0.034	121	31.0	8.00	15	0.327
Ventral	54.5	12.4	3.19	15		107	45.1	11.6	15	
Flash sensitivity, Sf (μV photon^−1^ μm^2^)										
Dorsal	0.22	0.10	0.03	15	0.044	0.073	0.027	0.007	15	0.574
Ventral	0.15	0.08	0.02	15		0.079	0.034	0.009	15	
Fractional Sensitivity, S_f_^b^ (photons^−1^ μm^2^)										
Dorsal	0.0147	0.0058	0.0015	15	0.014	0.0042	0.0014	0.00037	15	0.158
Ventral	0.0102	0.0031	0.0008	15		0.0055	0.0030	0.00078	15	
Time to peak, T_p_ (ms)										
Dorsal	145	28.2	7.29	15	0.458	302	182	47.1	15	0.010
Ventral	138	22.3	5.77	15		157	89.9	23.2	15	
Integration time, T_int_ (ms)										
Dorsal	256	65.0	16.8	15	0.585	940	602	156	15	0.010
Ventral	242	70.3	18.1	15		429	382	98.6	15	
Recovery time constant, τ_rec_ (ms)										
Dorsal	201	56.3	14.5	15	0.418	940	767	198	15	0.043
Ventral	223	87.4	22.6	15		442	490	127	15	

SD, standard deviation; SE, standard error; P, Student’s *t* test values for the indicated pairs of parameters in the corresponding genotype.

a Suction electrode recordings were performed as described in Experimental procedures; n = 15 rods from three mice of each indicated genotype.

b Fractional S_f_: S_f_ divided by R_max_.

**Figure 7:**
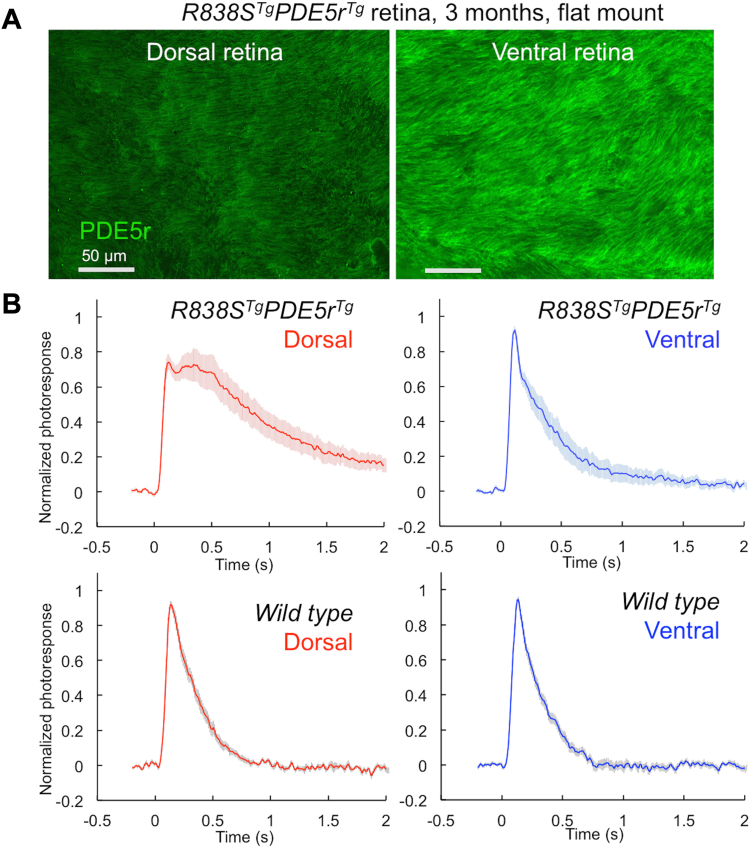
**The shape of photoresponse in rescued *R838S***^***Tg***^***PDE5r***^***Tg***^**rods varies depending on the efficiency of expression of PDE5r.***A*, flat-mount images of *R838S*^*Tg*^*PDE5r*^*Tg*^ retina probed with anti-FLAG antibody. The confocal images were taken at the same excitation and acquisition settings. PDE5r immunofluorescence is more robust in ventral part of the *R838S*^*Tg*^*PDE5r*^*Tg*^ retina. *B*, average normalized dim flash responses (mean ± SE) averaged from 15 dorsal (*red traces*) and ventral (*blue traces*) *R838S*^*Tg*^*PDE5r*^*Tg*^ (*upper* two panels) and WT rods (*lower* two panels) rods. PDE, phosphodiesterase; PDE5r, recombinant PDE5.

We further also observed that the shape of rod responses evidently reflected the relative efficiency of the PDE5r expression across the *R838S*^*Tg*^*PDE5r*^*Tg*^ retinas (Fig. 7; Table 1). When probed for expression of PDE5r, ventral retina displayed a higher intensity of the PDE5r immunofluorescence as compared to the dorsal half (Fig. 7*A*). Consistently with the differences in PDE5r expression, the rescued *R838S*^*Tg*^*PDE5r*^*Tg*^ rods from the dorsal *versus* ventral half of the retina displayed longer time-to-peak (T_p_ = 302 *versus* 157 ms; *t* test *p* = 0.01), longer integration time (T_int_ = 940 *versus* 429 ms; *p* = 0.01) and slower recovery kinetics (τ_rec_ = 940 *versus* 442 ms; *p* = 0.043) indicating more pronounced residual deregulation of calcium-feedback on R838S RetGC activity (12) in dorsal rods (Fig. 7*B*). Different parameters of photoresponse in *R838S*^*Tg*^*PDE5r*^*Tg*^ varied rod-to-rod, but on average appeared less different from the WT in the ventral retina (Fig. S1). The overall shape of the normalized dim flash response in *R838S*^*Tg*^*PDE5r*^*Tg*^ rods in the ventral retina was, except for some delay in reaching the full recovery, much more similar to that of WT rods (the latter did not substantially differ between the two parts of the retina) (Fig. 7*B*; Table 1).

### PDE5r rescues rods expressing Y99C GCAP1

**Figure 8:**
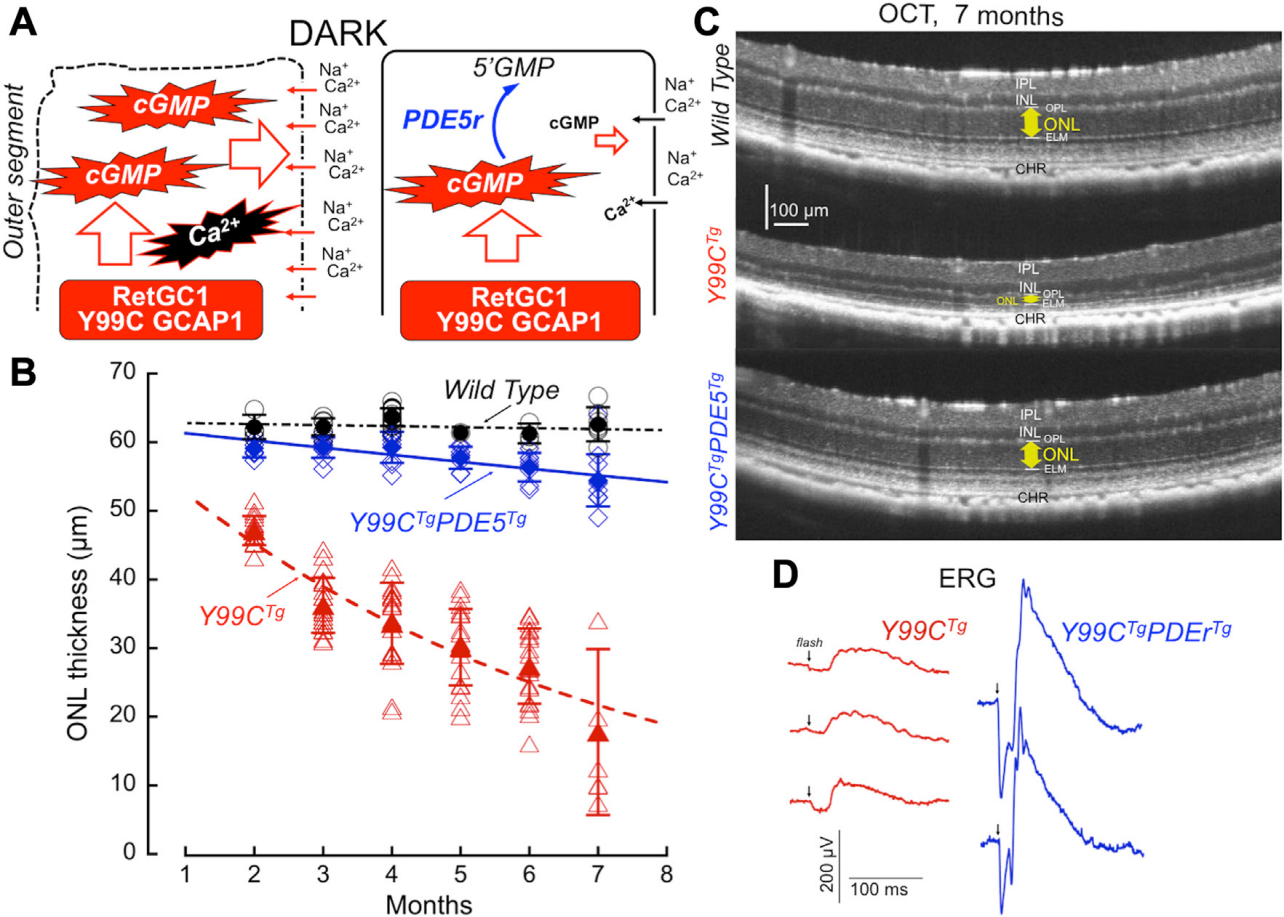
**PDE5r expression offsets rod degeneration in mice harboring Y99C GCAP1.***A*, the diagram of the expected change in cGMP flux in mouse rods harboring Y99C GCAP1 and hybrid rods harboring both Y99C GCAP1 and PDE5r. Deregulation of cGMP synthesis by the mutant Y99C GCAP1 at normal concentrations of Ca^2+^ in the dark elevates cGMP levels and increases influx of Ca^2+^ through the cGMP gated channels, provoking degeneration of *Y99C*^*Tg*^ rods (10, 11). *B*, photoreceptor nuclei layer thickness at different ages in WT (● ◯), *Y99C*^*Tg*^ ( ), and *Y99C*^*Tg*^*PDE5r*^*Tg*^ ( ) mice; *open symbols*–OCT measurements in different animals at the indicated ages; *filled symbols with error bars* (mean ± SD)–averaged ONL thickness at these ages; the differences between *Y99C*^*Tg*^*PDE5r*^*Tg*^ and *Y99C*^*Tg*^ were statistically significant (*t* test *p* < 0.0001) at all tested ages. *C*, representative *in vivo* OCT imaging of WT (*top*), Y99C^Tg^ (*middle*), and *R838S*^*Tg*^*PDE5r*^*Tg*^ (*bottom*) mice aged 7 months. The *yellow arrow* marks the thickness of ONL. *D*, bright-flash ERG (∼0.5 × 10^6^ R∗ rod^−1^) responses in different mice from the same litter containing three *Y99C*^*Tg*^ (*red traces*) and two *Y99C*^*Tg*^*PDE5r*^*Tg*^ (*blue traces*) 7-months old siblings. ERG, electroretinography; GCAP, guanylyl cyclase activating protein; OCT, optical coherence tomography; ONL, outer nuclear layer; PDE, phosphodiesterase; PDE5r, recombinant PDE5.

We explored the same paradigm for rescuing rods overproducing cGMP in the dark due to deregulation of the RetGC1:GCAP1 complex activity, this time because of a mutation in GCAP1, the main calcium-sensor of RetGC1 (13). Similarly to the *R838S*^*Tg*^, in a previously characterized transgenic mouse model expressing Y99C GCAP1 under control of rhodopsin promoter (*Y99C*^*Tg*^, line 52) (10, 11, 13), rods undergo degeneration triggered by deregulation of Ca^2+^ feedback on cGMP production in the dark, resulting in elevated free cGMP and Ca^2+^ levels in ROS (10, 11). Hence, we reasoned that PDE5r expression in *Y99C*^*Tg*^ rods could counteract the elevated production of cGMP in the dark (Fig. 8*A*) and thus protect *Y99C*^*Tg*^ rods *via* the same mechanism as *R838S*^*Tg*^ rods. The *Y99C*^*Tg*^ rods in that model degenerated slower than *R838S*^*Tg*^ and the extent of degeneration at different ages also varied more widely, but by 7 months of age the vast majority of the rods were eliminated (Fig. 8, *B* and *C*). In contrast, *PDE5^Tg^Y99C^Tg^* retinas displayed a dramatic preservation of the photoreceptor layer (Fig. 8, *B* and *C*). In one litter aged 7 months, ERG responses were tested and were much better preserved in *R838S*^*Tg*^*Y99C*^*Tg*^ than in their *Y99C*^*Tg*^ littermates, who produced only rudimentary ERG responses to a bright flash (Fig. 8*D*).

## Discussion

### The paradigm for rescuing GUCY2D and GUCA1A dominant retinopathies

Based on previous animal studies (36, 37) and the clinical cases describing loss of function in RetGC1 associated with a recessive blindness, *GUCY2D* Leber’s congenital amaurosis (38, 39, 40), RetGC1 is the main isozyme of RetGC providing most of cGMP production in mammalian photoreceptors, including human rods and cones. Studies in transgenic mouse models also show that RetGC1 isozyme can be regulated by GCAP1 and GCAP2, whereas the ancillary rod isozyme RetGC2 is regulated *in vivo* predominantly by GCAP2 (13). Certain rare forms of congenital *GUCY2D* night blindness may indicate that some individuals deviate from this more common pattern (41, 42). Nevertheless, the RetGC1:GCAP1 complex clearly plays the key role in pathogenesis of a different form of blindness, in which the cause of the disease is gain-of-function, abnormally high deregulated activity of cGMP production in the dark (9, 16, 43, 44).

Multiple cases of dominant cone- and cone-rod degenerations have been linked to various mutations in *GUCY2D* and *GUCA1A* coding for the respective RetGC1 and GCAP1 in human patients (9, 15, 16, 17, 18, 19, 20, 21, 22, 43, 44). The biochemical reasons that the complex RetGC1:GCAP1 becomes less sensitive to the deceleration by Ca^2+^ at the concentrations typical for dark-adapted photoreceptors are different in case of RetGC1 and GCAP1 mutants. RetGC1 mutations linked to the dominant retinopathy can reduce the sensitivity of the RetGC1:GCAP1 for Ca^2+^ by stabilizing the complex in which RetGC1 preferentially binds Mg^2+^GCAP1. This hinders Mg^2+^GCAP1 replacement by Ca^2+^GCAP1, the form that decelerates RetGC1, which requires higher than normal concentrations of Ca^2+^ to suppress cGMP production by RetGC1:GCAP1 in the dark (17, 18, 42). In contrast, mutations in GCAP1 typically directly or indirectly affect its EF-hands (45) affinity for Ca^2+^, which also results in incomplete deceleration of the RetGC1:GCAP1 complex activity at normal dark-adapted free Ca^2+^ concentrations (5, 9, 10, 11, 13, 15, 20, 21, 22).

Despite the differences in the actual molecular events that affect Ca^2+^ sensitivity of the cGMP production between *GUCY2D* and *GCAP1* dominant retinopathies, previous studies demonstrated that in both cases deregulation of cGMP production in the dark is the primary trigger for degeneration of photoreceptors harboring mutant RetGC1 or GCAP1 in living photoreceptors (11, 12, 13). It was previously observed that whereas rearing mice harboring the deregulated cGMP synthesis in complete darkness can exacerbate the degeneration (46), rearing them under constant light had very little, if any, benefit in reducing the severity of degeneration compared to rearing such mice in normal 12 h/12 h light/dark cycle (11). A permanent activation of PDE6 cascade in mouse rods can be achieved by using transgenic expression of G90D rhodopsin, which activates constitutively the Gt-PDE6 cascade in the dark and produces “equivalent light” activity of ∼130 photons/rod/sec (47, 48). This constitutive activity dramatically rescued the *Y99C*^*Tg*^ and *E155G GCAP1*^*Tg*^ rods from degeneration caused by deregulation of cGMP synthesis in the dark (11). However, the use of G90D rhodopsin to rescue dominant photoreceptor dystrophy faces two major, insurmountable obstacles. First, activation of PDE6 in the dark heavily desensitizes rods, such that they severely lose their light-sensitivity to dim light (11). Second, and even more importantly, G90D rhodopsin, just like the normal rhodopsin, is a membrane protein, the major component of a photoreceptor disk membrane. Therefore, its overexpression can compromise the integrity of the disk membrane and cause photoreceptor degeneration (47).

To overcome these obstacles, here we tested a different approach to reduce the impact of elevated cGMP production in the dark by ectopic expression of a nonphotoreceptor phosphodiesterase PDE5 in photoreceptors affected by the presence of RetGC1 or GCAP1 mutants that trigger retinal degeneration. One of the reasons for choosing PDE5 instead of trying to overexpress PDE6 was its relatively low catalytic activity, 5 to 10 fold lower than fully activated PDE6 (25, 26), which could lower the extent of desensitization compared to the effect of permanent activation of PDE6 (11). Another important reason was that PDE6 undergoes a rather complex chaperoning process that we could potentially disturb by overexpressing PDE6. Interfering with the PDE6 chaperoning can by itself cause photoreceptor cell death (28, 29, 30, 31, 32). Last but not least, PDE6, and especially rod PDE6, is a multisubunit enzyme that requires displacement of its inhibitory subunit by GTP-Gtα for its activation (24, 25, 26, 27, 28, 29). In contrast, PDE5 activity does not involve self-inhibition, nor does it require G protein–dependent activation (24, 25). We further reasoned that the use of a homodimeric PDE5 would simplify the prospects of gene delivery in potential gene therapy applications down the road.

### Ectopically expressed PDE5 accumulates in the outer segment

We considered that the main obstacle for the use of PDE5 was achieving its accumulation in the outer segment. PDE5 lacks the isoprenylation signal necessary for delivery to the outer segments that both rod and cone PDE6 catalytic subunits harbor at their C termini (28, 33). This presented a challenge because the main trigger for the photoreceptor degeneration in *GUCY2D* and *GUCA1A* retinopathies starts in the outer segment, where the mutant RetGC1:GCAP1 complex produces excess of cGMP in the dark, subsequently causing opening of the excessive number of cGMP gated channels, thus elevating free cGMP and Ca^2+^ in the outer segment (10, 11, 12). In addition, the cells harboring the RetGC1:GCAP1 complex become more depolarized in the dark (10, 12), which may conceivably increase Ca^2+^ influx through the voltage-gated Ca^2+^ channels in the synaptic part of the cell (49), and thus further exacerbate the apoptotic process caused by deregulation of cGMP synthesis in the outer segment. Regardless of the possible pathways through which the degeneration unfolds, the excess of free cGMP production is its primary trigger because deletion of GCAPs in rods harboring mutant RetGC1 (12) or deletion of RetGC1 in rods harboring mutant GCAP1 (13) rescue photoreceptors from degeneration. So, we reasoned that ectopically expressed PDE5 needed to be delivered specifically in the outer segment. Adding a short peptide sequence derived from a human PDE6Cα carrying isoprenylation “CAAX” box was sufficient to help accumulate the expressed PDE5 in the outer segment, where it locates internally to the plasma membrane. It remains to be established in future studies how PDE5 interacts with the disk membranes, but most likely it associates with the stack of the photoreceptor disks (Fig. 2, *D* and *E*), similarly to PDE6, a soluble protein in ROS associated with the photoreceptor disks as a disk membrane peripheral protein of phototransduction cascade (1, 2, 3, 24, 25, 26, 27, 28, 29). Evidently, the isoprenylation signal on PDE5r derived from PDE6C not only improved the general ability of PDE5 to associate with membranes (Fig. 1*B*), but also allowed the rod cell trafficking mechanisms to accumulate PDE5r in the stack of disk membranes of the outer segment.

### Photoreceptor survival improves at the expense of light sensitivity

The dramatic rescue of degeneration caused by ectopic PDE5 expression (Figs. 4 and 8) is a trade-off in which preserving photoreceptors occurs at the expense of reduction of their light-sensitivity (Figs. 3 and 6; Table 1). The light sensitivity is evidently reduced by the activity of PDE5r in the dark (Fig. 3), which produces the “equivalent light” effect of ∼20 to 40 photons μm^−2^ sec^−1^. This reduces relatively mildly light sensitivity of rods as compared to the effect of PDE6 activation by G90D rhodopsin (11, 47, 48). Overall, the light sensitivity of the rescued rods is still preserved much better than in the retinas undergoing degeneration. This mild elevation of PDE activity in the dark is evidently sufficient to counteract the deregulated cGMP production and preserve most of the light sensitivity by rescuing rods from degeneration (Fig. 6). It was quite interesting that the shape of the photoresponse in rescued rods was different from the WT (Figs. 7, S1, and Table 1), showing longer integration time and slower recovery phase. The cell-to-cell and dorsal *versus* ventral variability of PDE5r expression (Fig. 7*A*) makes the variability of the responses much higher than in WT (Figs. 7*B* and S1) because the extent of the changes evidently depends on the levels of PDE5 expression *versus* that of R838S RetGC1. In the ventral rods, which express PDE5r more robustly (Fig. 7), the responses were more similar to the WT, whereas the rescued dorsal rods, with lower PDE5r expression displayed a more pronounced residual deregulation of the Ca^2+^ feedback. So, even though those rods are rescued, Ca^2+^ levels in some of them likely remain above normal, which in combination with the less cooperative Ca^2+^ feedback on the cyclase (10, 12, 34) can make the cGMP acceleration a rate-limiting factor in recovery, even in the conditions when the dark activity of PDE5 reduces the abnormally elevated free cGMP synthesis and limits the increase of Ca^2+^ to the levels triggering apoptosis. This also brings a question, how far the cGMP and Ca^2+^ levels need to elevate in order to trigger degeneration, and what levels photoreceptors can still tolerate without succumbing into apoptosis. At this point, we do not have a clear answer to that question.

### Possible perspectives for using ectopic PDE expression for gene therapy of dominant GUCY2D and GUCA1A retinopathies

The rescue of degenerating rods in mouse models in this study is quite remarkable (Figs. 4 and 8), so the prospect of using PDE5r for gene therapy deserves to be further evaluated. Whereas our study provides a general proof-of-concept for using this approach, the main challenge for its potential clinical application remains to be tested starting with it is effectiveness in preventing cone degeneration. Currently, we do not have models that would adequately mimic cone dystrophy caused by the mutant RetGC1 or GCAP1. Developing such models could provide a more definitive answer to that question.

The next step in evaluating the potential application of PDE5r for gene therapy would also require a study in which PDE5r is delivered by parvovirus particles (23). The size of the PDE5r cDNA is quite suitable for this approach. Other isozymes of PDE could also be explored for this purpose, but need to be tested first in animal model in comparison with PDE5r. For example, we also attempted to use cGMP-specific PDE9A5 (27), a splice variant of PDE9 (NCBI: NM_001001570.1) almost 40% shorter than PDE5A polypeptide and therefore potentially suitable for delivery as self-complimentary adeno-associated viral vector. However, we observed that in contrast to PDE5r the PDE9r protein precipitated into massive aggregates in HEK cells and inner segments of *PDE9r*^*T*g^ rods (data not shown), so we excluded this particular isozyme from further consideration. Nonetheless, testing other PDE isozymes and splice variants in transgenic models could provide viable options along with the use of PDE5r.

Could PDE5r be used in other types of retinal degenerations induced by cGMP elevation when such elevation is caused by deficiency of PDE6 rather than by deregulation of RetGC? We do not think that the use of PDE5r would be a good strategy for rescuing rods completely lacking PDE6, because PDE5 is not a part of phototransduction. In case of complete deficiency of PDE6, the expression of the corresponding normal PDE6 subunit would be a better choice for the prospective gene replacement therapy. Yet, it is not inconceivable that in some situations, such as when PDE6 is partially lost due to abnormal overload of its chaperoning complexes by a mutant PDE6 subunit, PDE5 could be used to reduce, without aggravating the burden on the PDE6 chaperoning system, the cGMP levels while still allowing the residual PDE6 to perform its function in phototransduction. Such a possibility would likely be feasible to evaluate in animal studies.

## Experimental procedures

### Animals

All experiments involving animals were conducted in accordance with the Public Health Service guidelines and approved by the Drexel University and University of California Irvine Institutional Animal Care and Use Committees. The WT C57BL/6J mouse strain originated from JAX Research/Jackson’s Laboratory. *R838S*^*Tg*^ mice (line 379) and Y99C^Tg^ (line 52) were produced as described previously (10, 12, 34) and made congenic to the C57BL/6J background by repetitive breeding for over 10 generations prior to conducting the experiments. Mice expressing PDE5r (‘line 22') were produced in C57BL/6J background by random insertion of a PDE5r-coding construct *via* mouse egg injection service provided by Cyagen/Taconic. Mice were fed the same diet and were housed in the same temperature- and humidity-controlled environment using 12 h/12 h light/dark cycle.

### PDE5r transgene construct

Mouse Pde5a-tagged cDNA clone inserted in pCMV6 vector was purchased from OriGene (cat#MR225179). A synthetic (Integrated DNA Technologies) DNA fragment coding for two FLAG epitopes and eight C-terminal residues of a human PDE6C alpha subunit containing its isoprenylation “CAAX box” signal (25, 33), CLML (Fig. 1*A*), 5′-ACGCGTGGAGGCGGAGGTGGAGCTAGCGGAGGAATCGATGATTACAAGGATGACGACGATAAGAAGTCCAAAACATGTTTAATGTTGTAAGCGGCCGCGG, was inserted into the MluI/SacII sites of the pCMV6Pde5a plasmid to construct PDE5r cDNA. The PDE5r coding fragment derived from the resultant pCMV6PDE5r construct was ultimately inserted into a Stratagene pBluescript plasmid containing a 4.2-kbp mouse rhodopsin promoter region and a 0.5-kbp fragment of the last exon of a mouse protamine gene containing polyadenylation signal (50) (Fig. 2*A*). The *PvuI/XbaI* fragment excised from the plasmid was injected in male pronuclei of fertilized C57BL/6J mouse eggs (a service from Taconic/Cyagen) to develop F_0_ founders. The founders were genotyped by PCR using tail DNA samples and two primers, 5′- CCTGACCCACGTATCCGAAGACTGTTT and 5′- CATCTGCTCCTGCTTTTGCTGCGG. One of the three founders (F_0_22) passed the transgene to progeny and that line (‘line 22’) was subsequently bread to C57BL/6J, *R838S*^*Tg*^ line 379 (34), and *Y99C*^*Tg*^ line 52 (10) mice to generate the respective *PDE5r*^*Tg*^*, R838S*^*Tg*^*PDE5r*^*Tg*^, and *Y99C*^*Tg*^ *PDE5r*^*Tg*^ lines. The presence of the R838S RetGC1 and Y99C GCAP1 coding transgenes in mouse tail DNA samples for genotyping was detected as previously described (10, 34).

### Expression of PDE5r in HEK cells

PDE5r was first expressed from the pCMV6PDE5r vector using a Promega FuGENE protocol in HEK 293 cells (originated from Invitrogen) on Lab-Tek 4-well cover glass, transfected after reaching 30∼50% confluence when incubated at 37°, 5% CO2. The expression was confirmed using a Sigma-Aldrich (Millipore) mouse monoclonal anti-FLAG antibody after paraformaldehyde fixation of the cultures (Fig. 1*B*). The live cells prior to fixation were additionally counterstained for 30 min at 37°, 5% CO_2_, with 1 μM endoplasmic reticulum-tracker Red reagent (MedChem Express, catalog# M2501474) diluted in a Molecular probes Live cell imaging solution from 1000x stock in dimethyl sulfoxide. To test the presence of PDE5r activity, the pCMV6PDE5r plasmid was transfected using polyethyleneimine (PEI) Max-40 protocol (51) in suspension culture of FreeStyle293 cells grown in FreeStyle293 medium (Invitrogen/Thermo Fisher Scientific). The 30-ml suspension cell culture was grown in 125-ml culture flasks rotated at 130 rpm at 37 °C in a CO_2_ incubator at 8% CO_2_ to a density of 1 × 10^6^ cells/ml (≥ 95% viable cells). For the transfection, 60 μl of 1 μg/μl PEI Max-40 was mixed with 0.6 ml OptiPRO SFM reagent (Invitrogen/Thermo Fisher Scientific) and then mixed with 30 μg of pCMV6PDE5r plasmid DNA in 0.6 ml OptiPRO SFM. pCMV6PDE5r-transfected FreeStyle cells were harvested 48 h post transfection at ∼2 × 10^6^ cells/ml by centrifugation for 20 min at 4000 rpm in a Sorvall Legend RT centrifuge, at 4 °C. The cell pellet was resuspended in 0.6 ml Tris-buffered saline (TBS), pH 7.4 (Thermo Fisher Scientific), containing 1:100 dilution of protease inhibitors cocktail (Sigma-Aldrich/Millipore), aliquoted in 50-μl samples, frozen in liquid nitrogen and stored in −70 °C. The nontransfected control cells were harvested using the same procedure.

### PDE activity assay

PDE activity was assayed in 25 μl reaction mixture containing 55 mM Tris–HCl, pH 7.9, 25 mM NaCl, 0.5 mM KCl, 2.5 mM MgCl_2_, 2 mM cGMP (Sigma-Aldrich/Millipore), ∼0.2 μCi [8-^3^H]cGMP (PerkinElmer), 1 mM 5′GMP (Sigma-Aldrich/Millipore), 25 μg/ml leupeptine (Sigma-Aldrich/Millipore), and 3 μl homogenized FreeStyle 293 cell frozen/thawed suspension. The samples were incubated for 10 min at 30 °C, inactivated for 2 min at 95 °C, and centrifuged for 10 min at 14,000 rpm in an Eppendorf-5417C centrifuge at room temperature. A 5-μl aliquot from the supernatant was loaded on a fluorescent plastic-backed PEI cellulose plate (Merck) and developed first in water and then in 0.3 M LiCl. The cGMP and 5′GMP spots were visualized under UV illumination, cut out of plate and placed in 20-ml scintillation vials. The ^3^H radioactivity from the spots was eluted in 0.5 ml of 2 M LiCl and counted in 10 ml UniverSol scintillation cocktail (MP Biomedical). To measure the PDE activity in mouse retinas, the dark-adapted overnight mice were euthanized in the dark and the retinas from enucleated eyes were dissected under infrared illumination using night vision goggles in 20 μl/retina of TBS containing 2.5 mM MgCl_2_ and 1:100 dilution of protease inhibitors. The harvested retinas were homogenized after adding 50 μl/retina of the same buffer containing 6 mM 2-mercaptoethanol, the homogenates were aliquoted in 60-μl samples, wrapped in aluminum foil, frozen in liquid N_2_ and stored at −70 °C. The concentration of rhodopsin in the samples was measured by *A*_500_ absorbance. An aliquot of the homogenate was mixed with equal volume of TBS buffer containing 2.3% N,N-dimethyl-n-dodecylamine N-oxide and 2 mM hydroxylamine, incubated 5 min in the dark at room temperature. The mixture was centrifuged for 5 min at 10,000*g*, 4 °C to remove the remaining debris, and the absorbance of solubilized rhodopsin in the supernatant at 500 nm was measured immediately before and after complete bleaching by white light. The concentration of rhodopsin was calculated assuming ε∼40,000 M^−1^ cm^−1^ at 500 nm and molecular mass 39 kDa (ca. 1 mg/ml rhodopsin/1 optical unit *A*_500_). PDE activity was assayed as described above except that instead of FreeStyle 293 cells, the reaction mixture contained 0.25 μg rhodopsin/assay in retinal homogenates. The incubation time for measuring PDE activity in the dark in the absence of guanylyl imidodiphosphate was 20 min at 30 °C, whereas in the light in the presence of guanylyl imidodiphosphate it was reduced to 5 min to maintain the linear time-course of cGMP hydrolysis.

### RetGC activity assays

The mouse retina homogenates were prepared in the dark as described above. The retinas for RetGC activity measurements were excised from the dark-adapted 3.5-weeks old mice using infrared illumination (Kodak number 11 infrared filters) and a dissecting microscope fitted with an excalibur infrared goggles, wrapped in aluminum foil, frozen in liquid N_2_, and stored at −70 °C prior to their use in the cyclase activity assays, also conducted under infrared illumination. The guanylyl cyclase activity was assayed as previously described in detail (34). In brief, the assay mixture (25 μl) containing retinal homogenate in 30 mM Mops–KOH (pH 7.2), 60 mM KCl, 4 mM NaCl, 1 mM DTT, 2 mM Ca^2+^/Mg^2+^/EGTA buffers, 0.9 mM free Mg^2+^, 0.3 mM ATP, 4 mM cGMP, 1 mM GTP, and ∼1 μCi of [α–^32^P]GTP, 100 μM Zaprinast and dipyridamole, and 10 mM creatine phosphate/0.5 unit of creatine phosphokinase (Sigma-Aldrich/Millipore) was incubated at 30 °C for 12 min, and the reaction was stopped by heat-inactivation at 95° for 2 min. The resultant [^32^P]cGMP product was separated by TLC using fluorescently backed polyethyleneimine cellulose plates (Merck) developed in 0.2 M LiCl, cut from the plate, eluted with 0.5 ml of 2 M LiCl in scintillation vials, and the radioactivity was counted using liquid scintillation. The assay contained ∼0.5 μCi [^3^H]cGMP internal standard to ensure the lack of the cGMP product hydrolysis by retinal PDE. Ca^2+^/EGTA buffers at 0.9 mM free Mg^2+^ were prepared using Tsien and Pozzan method (52) and verified by fluorescent indicator dies as previously described in detail (53). Data fitting, a=(amax−amin)/(1+([Ca2+]free/Ca122+)n)+amin, where *a* is the activity of RetGC, *a*_*max*_ and *a_min_* are the respective maximal and minimal activity, Ca^2+^_1⁄2_ is the free Ca^2+^ concentration at half-maximal inhibition, and *n* is the cooperativity coefficient, was performed using Synergy KaleidaGraph 4 software (https://www.synergy.com).

### Retinal postmortem morphology

Mice were anesthetized with a lethal dose of ketamine/xylazine injection, perfused through the heart with PBS and then with 2.5% glutaraldehyde/3% paraformaldehyde (Electron Microscopy Sciences) in PBS. The eyes were surgically removed and fixed overnight in 2.5% glutaraldehyde/3% paraformaldehyde in PBS at room temperature. The fixed eyes were washed in PBS, soaked in PBS overnight, processed for paraffin embedding, sectioned at 5-μm and stained with hematoxylin/eosin (AML Laboratories). The retinal sections were photographed using an Olympus BX21 microscope fitted with an Olympus MagnaFire camera.

### Optical coherence tomography

Mice were anesthetized using intraperitoneal injection of ∼20 mg/kg ketamine and ∼8 mg/kg xylazine (Penn Vet). The pupils were dilated by applying 1% tropicamide and 2.5% phenylephrine ophthalmic eye drops ∼5 to 10 min before the scan. The A-scans and then B-scans of the retinas were acquired using an IISCIENCE OCT camera calibrated by the manufacturer at 2.47 μm/pixel axial scale and 3.5 μm/pixel lateral scale resolution and averaged from ∼200 frames. The thickness of the outer nuclear layer was measured between the outer plexiform and the external limiting membrane reflective layers (54, 55) from the B-scans made across the ventral part of the retina, ca. 400 to 700 μm below the optic nerve.

### Antibodies

Anti-GCAP1 (RRID: AB_3668998), anti-GCAP2 (RRID: AB_3668997), anti-recoverin (RRID: AB_3668996) anti-RetGC1 (RRID:AB_2877058) rabbit polyclonal antibodies were characterized previously and validated using transgenic mouse models (56, 57, 58, 59); rabbit polyclonal anti-peripherin 2 (Prhp2) antibody was a generous gift from Dr Andrew Goldberg (Oakland University), anti-FLAG mouse monoclonal antibody (F3165) was purchased from Sigma-Aldrich (Millipore). Polyclonal mouse antibody against CNG1 alpha subunit (RRID: AB_3669045) was produced and validated as described previously (14), anti-PDE5A (ab259945) and anti-PDE6A (ab5659) antibodies were purchased from Abcam, anti-GRK1 antibody was a generous gift from Dr Ching-Kang Jason Chen (University of Texas at San Antonio) and anti-Gtα (sc-389) antibody was purchased from Santa Cruz Biotechnology. Commercial antibodies were validated by the manufacturers.

### Immunoblotting

The retina homogenates from mice equilibrated by rhodopsin content as described above were subjected to immunoblotting after extraction in an Abcam radioimmunoprecipitation assay mixture of detergents containing protease inhibitors cocktail (Millipore/Sigma). The samples mixed 1.5 volume of 5 × Laemmli SDS sample buffer (Millipore/Sigma) were subjected, next to a set of prestained PageRuler Plus molecular mass markers (Pierce/Thermo Fisher Scientific), to electrophoresis in 4 to 20% gradient polyacrylamide gel (Invitrogen/Thermo Fisher Scientific) using running buffer containing 0.1% SDS. Following the electrophoresis, the proteins were transferred overnight at 50V constant voltage to Immobilon P membrane (Millipore) at 18 °C using Tris-glycine transfer buffer (Invitrogen/Thermo Fisher Scientific). The membrane was washed with Tris-buffered saline (Thermo Fisher Scientific) containing 0.5% Tween-20 (TTBS), blocked by SuperBlock (Thermo Fisher Scientific) solution in TTBS, and probed by primary antibody for 1 h at room temperature, followed by washing and probing with the secondary antibody. The secondary antibody was removed by washing three times x 15 min in TTBS and twice in TBS. The luminescence signal was developed using peroxidase-conjugated secondary polyclonal anti-rabbit and anti-mouse IgG (Cappel/MP Biomedical) and a Pierce SuperSignal Femto substrate reagent (Thermo Fisher Scientific). The images were acquired and processed using a Fotodyne Luminous FX imager and ImageJ (National Institutes of Health) software (https://imagej.net/software/imagej/).

### Confocal microscopy

Mice were euthanized by lethal injection of ketamine/xylazine and perfused with formaldehyde fixative solution, 1% for flat-mount staining and 5% for cryosectioning. Then the retinas for flat mount were excised and fixed in 4% paraformaldehyde for subsequent staining with the anti-PDE5A or anti-FLAG antibody to label PDE5r. For cryosections, the whole enucleated eyes were dissected using cryo microtome, and the sections were mounted for microscopy as previously described (14, 34). The whole retinas for flat-mount images and the cryosections were washed three times in PBS containing 0.1 M glycine (pH 7.4), blocked for 1 h at 30 °C with the same solution containing 5% bovine serum albumin and 0.1% Triton X-100, incubated overnight at 4 °C and then 1 h at room temperature with the primary antibody, then washed with PBS solution three times for 15 min each, incubated with diluted anti-rabbit or anti-mouse secondary fluorescent antibody, and washed four times for 15 min with PBS at room temperature. Confocal images were acquired using an Olympus FV1000 Spectral instrument controlled by FluoView FV10-ASW software, collecting the emitted fluorescence of different wavelengths in a sequential mode. Where indicated, the antibody fluorescence was superimposed on a differential interference contrast image or TO-PRO-3 dye fluorescence. The far-red fluorescence of TO-PRO-3 in the images was assigned blue pseudo-color. No changes were made to the original images, except for gamma correction applied to the whole image for better clarity in print.

### Electroretinography

The mice were dark-adapted overnight, their pupils were dilated by applying 1% tropicamide and 2.5% phenylephrine ophthalmic eye drops under dim red safelight illumination, and the mice were dark-adapted for another 10 min. Full-field ERG in the mice anesthetized by inhalation of 1.7 to 1.9% isoflurane (VEDCO)/air mix delivered by a Kent Scientific SomnoSuite setup at 50 ml/min was performed in the dark as previously described in detail (14, 34) using a Phoenix Research Laboratories Ganzfeld ERG2 instrument. Light pulses producing 0.5 to ∼0.5 × 10^6^ R∗ rod^−1^ (per manufacturer calibration) were injected into the eye through the infrared camera-guided corneal electrode/LED light source of the instrument.

### Suction electrode recordings

Single-cell suction recordings were performed as described previously (12). The mice were dark-adapted overnight and euthanized by CO_2_ inhalation and subsequent cervical dislocation. Retinas were dissected into Locke’s solution (112.5 mM NaCl, 3.6 mM KCl, 2.4 mM MgCl_2_, 1.2 mM CaCl_2_, 10 mM Hepes, pH 7.4, 20 mM NaHCO_3_, 3 mM sodium succinate, 0.5 mM sodium glutamate, 0.02 mM EDTA, and 10 mM glucose, 0.2% (v/v) minimum essential medium amino acids (50x) solution (M5550, Sigma-Aldrich), and 0.1% (v/v) minimum essential medium Vitamin solution (M6895, Sigma-Aldrich), pH 7.4 by bubbling with 95%O_2_/5%CO_2_) using a pair of micro scissors and fine forceps under a stereomicroscope with an infrared illuminator and infrared scope. The retina was cut into small pieces randomly by a razor blade for about 70 times and transferred into a custom-made perfusion chamber placed on the stage of an inverted microscope (IX51, Olympus). Warm Locke’s solution (1.2 ml/min, 35–37 °C) was continuously perfused by gravity during the recordings. The retinal fragments and recording pipette were monitored by camera under infrared illumination and displayed on a liquid crystal display. Recording and reference pipettes were made as described previously (60, 61). The recording pipette was heat-polished to obtain 1.5 to 2 μm inner diameter which fits well with an individual rod outer segments. A rod outer segment was drawn into the pipette by applying negative pressure from mouth to the electrode holder *via* oil-filled tubing, and stimulated with 500 nm flashes delivered from a custom-made light-emitting diode system (ref). Photon density of the flash was calibrated with an optometer (350 Linear/Log optometer, UDT Instruments) prior to the recordings. Photocurrent was amplified (Axopatch 200B, Molecular Devices), low pass filtered at 30 Hz (8 pole Bessel; Model 3382, Krohn-Hite), and imported to a computer through a digitizer (Digidata 1440A, Molecular Devices). Data acquisition and analysis were performed on Clampfit 10 (Molecular Devices; https://www.moleculardevices.com/).

### Statistics

Where applicable, normality of distribution was tested by Kolmogorov-Smirnov test; statistical significance of the differences was tested by ANOVA/Scheffe *post hoc* (confidence level 99%; alpha 0.01) or Student’s *t* test (unpaired) using Synergy Kaleidagraph or GraphPad software (https://www.graphpad.com/). The pertinent statistical differences are shown in Table 1 and in Results.

## Data availability

The data referred to in this manuscript are contained within the manuscript and the Supplemental figure and table. Unprocessed data can be available upon reasonable request from the corresponding author (alexander.dizhoor@drexel.edu).

## Conflict of Interest

The authors declare that they have no conflicts of interest with the contents of this article.

## Figures and Tables

**Supplement Fig S1:**