# Supplementary material for: Phosphodiesterase 5 expression in photoreceptors rescues retinal degeneration induced by deregulation of membrane guanylyl cyclase
Source: J Biol Chem. 2025 Feb 3;301(3):108265. doi: 10.1016/j.jbc.2025.108265 (PMC11923828; doi:10.1016/j.jbc.2025.108265)
Supplement: Supplement Figure legend [file mmc1.docx]

**Supplemental figure legend**

**Supplemental Fig. S1**. Distribution of parameters obtained from photoresponses of individual *WT* (*gray circles*) and *R838S^Tg^PDE5r^Tg^* (*blue circles*) rods in dorsal (D) versus ventral (V) parts of the retinas are shown superimposed on mean average (*thick horizontal bars*) ± SD (*error bars*) from 15 rods.
