# Supplementary material for: Phosphodiesterase 5 expression in photoreceptors rescues retinal degeneration induced by deregulation of membrane guanylyl cyclase
Source: J Biol Chem. 2025 Feb 3;301(3):108265. doi: 10.1016/j.jbc.2025.108265 (PMC11923828; doi:10.1016/j.jbc.2025.108265)
Supplement: Supplement Table S1 [file mmc2.docx]

**Supplemental Table S1***.*

Parameters of rod photoresponse in wild type and *PDE5r^Tg^* mice.

|  | R_max_ (pA) | | I_1/2_ (photon µm^-2^) | | S_f_ (pA photon^-1^ µm^2^) | | Fractional S_f_ (photon^-1^ µm^2^) | |
| --- | --- | --- | --- | --- | --- | --- | --- | --- |
| WT control, 25 rods | 14 ± 5 | | 33 ± 15 | | 0.30 ± 0.15 | | 0.021 ± 0.01 | |
| PDE5r^Tg^ , 30 rods | 16 ± 5.5 | | 75 ± 33 | | 0.14 ± 0.055 | | 0.0088 ± 0.0043 | |
| Mean ± SD.  **Supplemental Table S1 legend:** | |  | |  | |  | |  |

R_max_: estimated maximum response amplitude, I_1/2_: half-saturating flash intensity, S_f_: flash sensitivity, Fractional S_f_: S_f_ divided by R_max_
